# Endothelial-to-mesenchymal transition: An underappreciated mediator of diabetic complications

**DOI:** 10.3389/fendo.2023.1050540

**Published:** 2023-01-27

**Authors:** Eric Wang, Honglin Wang, Subrata Chakrabarti

**Affiliations:** Department of Pathology and Laboratory Medicine, Schulich School of Medicine and Dentistry, Western University, London, ON, Canada

**Keywords:** diabetes, diabetic complications, endothelial-to-mesenchymal transition, epigenetic regulation, vascular dysfunction

## Abstract

Diabetes and its complications represent a great burden on the global healthcare system. Diabetic complications are fundamentally diseases of the vasculature, with endothelial cells being the centerpiece of early hyperglycemia-induced changes. Endothelial-to-mesenchymal transition is a tightly regulated process that results in endothelial cells losing endothelial characteristics and developing mesenchymal traits. Although endothelial-to-mesenchymal transition has been found to occur within most of the major complications of diabetes, it has not been a major focus of study or a common target in the treatment or prevention of diabetic complications. In this review we summarize the importance of endothelial-to-mesenchymal transition in each major diabetic complication, examine specific mechanisms at play, and highlight potential mechanisms to prevent endothelial-to-mesenchymal transition in each of the major chronic complications of diabetes.

## 1 Introduction

Diabetes has long since reached epidemic levels around the globe and shows no signs of slowing down any time soon (1, 2). The current prevalence of diabetes has far surpassed projections made in the beginning of the century, and the number of people affected by diabetes is predicted to increase by nearly 50% by 2045 (3–5). Many people living with diabetes are affected by chronic diabetic complications, and with the increase in prevalence of diabetes, the burden of diabetic complications is sure to increase. Diabetes and its complications are significant causes of morbidity and mortality. Not only was diabetes directly responsible for 1.5 million deaths in 2019—making it the 9^th^ leading cause of death—diabetes is also a major risk factor for other leading causes of death such as heart disease, stroke, and kidney failure (1, 4).

The pathology of diabetic complications begins with vascular endothelial cells and eventually snowball into dysfunction of organs (6–8). Besides glycemic control, current approaches to management and treatment of these complications are focused on late-stage occurrences, and do not address the root problems (9–11). ln an attempt to shift the focus of conversations surrounding diabetic complications away from later stage outcomes and toward the early events and root causes, we examine the importance of endothelial-to-mesenchymal transition (EndMT), an early occurrence in diabetic complications that may represent a common target for prevention or management of different complications of diabetes.

### 1.1 Diabetic complications

Diabetic complications are broadly separated into two categories, micro- and macrovascular (12–14). As their names imply, microvascular complications affect capillaries and small vessels, while macrovascular complications affect larger vessels. Major microvascular complications of diabetes include diabetic retinopathy, diabetic nephropathy, diabetic neuropathy, and diabetic cardiomyopathy (12–14). Both micro- and macrovascular diabetic complications are primarily the result of hyperglycemia. Excessive amounts of circulating glucose is taken up by vascular endothelial cells (ECs), causing metabolic derangements within the ECs and resulting in damage (12–14).

ECs are among the first to be damaged by hyperglycemia during diabetes. Because ECs express the insulin-independent glucose transporter, GLUT1, their rate of glucose uptake is proportional to the amount of glucose in circulation (12–15). Excessive uptake of glucose overloads the glycolytic pathway, leading to the shunting of glycolytic intermediates into other, more harmful routes (12, 13). Furthermore, elevated rates of oxidative respiration downstream of increased glycolysis causes enhanced production of reactive oxygen species (ROS) in the mitochondria, leading to oxidative damage (12, 13). Oxidative damage triggers the activation of anti-ROS responses, one side-effect of which is the inhibition a glycolytic enzyme—GAPDH—which further compounds the shunting of glycolytic intermediates into harmful pathways (12, 13).

Harmful pathways activated by hyperglycemia include the polyol, hexosamine, protein kinase C (PKC) and advanced glycation end-product (AGE) pathways (12, 13). The polyol pathway reduces glucose into sorbitol at the expenditure of NADPH, an important cofactor in regenerating the antioxidant glutathione (12, 13, 16). Sorbitol can be oxidized into fructose, which can be fed back to the glycolytic pathway, but the depletion of NADPH increases susceptibility to damage by mitochondrial ROS (12, 13, 16). The hexosamine pathway shunts fructose-6-phosphate to generate uridine diphosphate N-acetyl glucosamine, which can covalently attach to transcription factors and disrupt gene expression (12, 13, 17). The PKC pathway involves PKC activation by diacylglycerol, converted from high levels of dihydroxyacetone phosphate, leading to widescale changes in gene expression (12, 13, 18). PKC activation in diabetes promotes angiogenic, fibrotic, and proinflammatory changes (12, 13, 18). Finally, the AGE pathway involves the non-enzymatic glycation of proteins by a variety of glucose-derived molecules, resulting in dysfunction of the glycated protein, and promoting inflammation by activating receptors of AGEs (RAGEs) (12, 13, 19).

The shunting of glucose into damaging pathways is a well-established part of hyperglycemia-induced endothelial dysfunction and occurs in large and small vessels alike. These pathways are activated early on during the pathogenesis of diabetic complications, but clinical manifestations of diabetic complications do not appear until much later. Thus, these occurrences alone do not paint the whole picture of early endothelial dysfunction and diabetic vascular complications.

### 1.2 Endothelial-to-mesenchymal transition

In response to specific stimuli, ECs can undergo a dramatic transformation known as EndMT. EndMT is a process whereby ECs, in response to specific internal and environmental triggers, transdifferentiate into mesenchymal like cells, losing their original endothelial characteristics and adopting mesenchymal phenotype (20–24). EndMT can be a physiological process and is a crucial occurrence in the embryonic development of heart valves (20–23). However, when aberrantly induced by environmental stressors such as hyperglycemia, EndMT gives rise to issues that contribute to dysfunction (21–24). The loss of endothelial junctional markers such as vascular endothelial cadherin (VE-CAD) and platelet endothelial cell adhesion molecule-1 (PECAM1) can lead to increased vascular permeability, resulting in increased infiltration of immune cells and unwanted exchange of fluids or factors between blood and the impacted tissue (25–27). While the gain of mesenchymal phenotype leads to increased production and deposition of extracellular matrix (ECM) proteins, contributing to sclerosis and fibrosis during later stages of disease (28–30).

**Figure 1:**
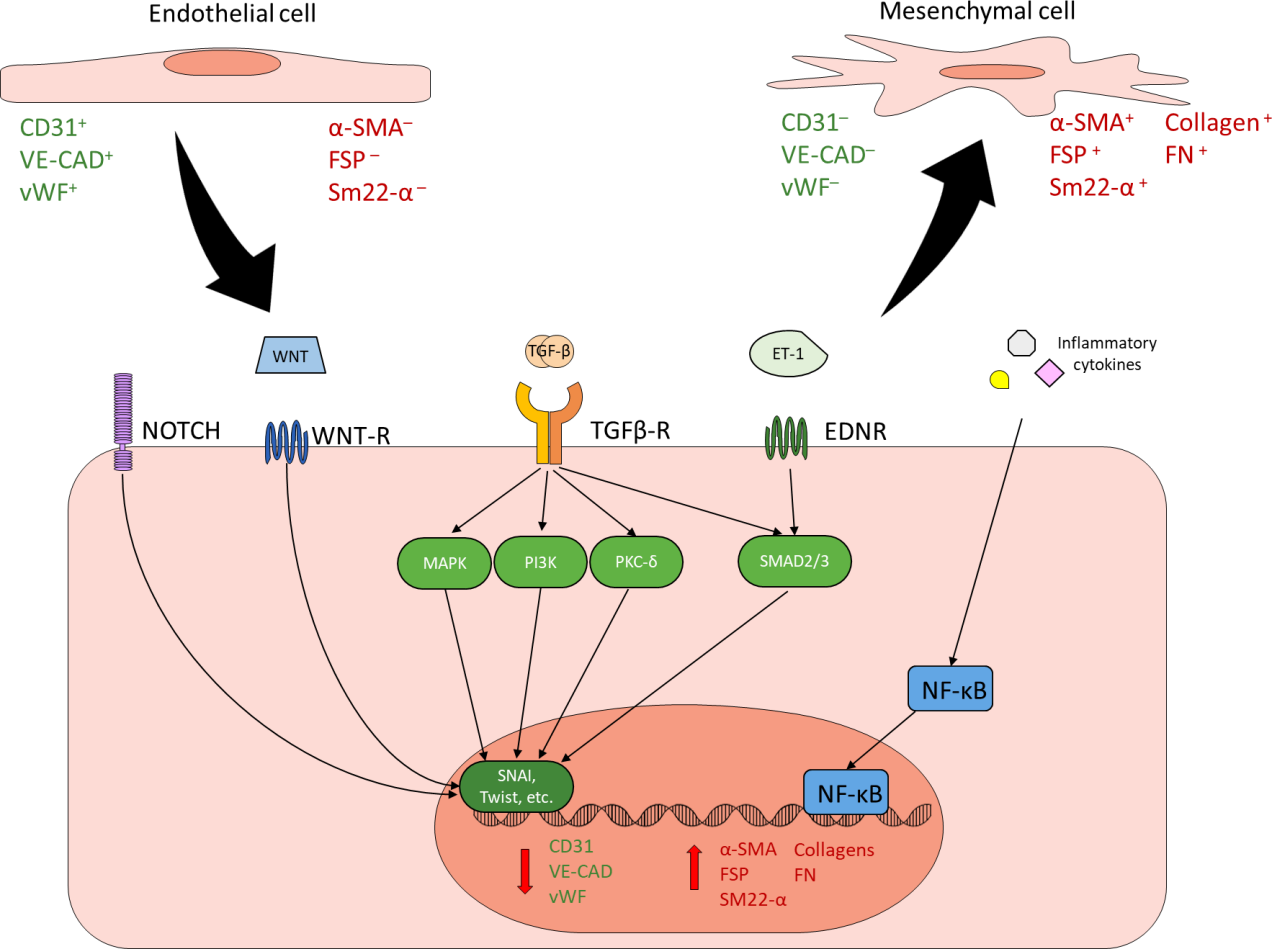
Endothelial to mesenchymal transition. ECs can undergo EndMT in response to a variety of stimuli. EndMT is characterized by reduced expression of endothelial markers (green) and increased expression of mesenchymal markers (red). Common inducers of EndMT include NOTCH, WNT, TGF-β, ET-1, and inflammatory signaling. TGF-β is the best characterized and most common driver of EndMT. Canonical TGF-β signaling through SMAD2/3, non-canonical TGF-β signaling through other mechanisms such as MAPK, PI3K and PKC-δ, as well as other EndMT inducers such as NOTCH, WNT, and ET-1 generally converge through the regulation of key transcription factors such as SNAI and Twist in order to reduce endothelial protein expression and promote mesenchymal protein expression. Inflammatory cytokines contribute to EndMT by activating NF-κB. Cells undergoing EndMT co-express endothelial and mesenchymal markers.

EndMT, whether physiological or pathological, is a regulated process mediated by specific extracellular signals and intracellular changes. Transforming growth factor β (TGF-β) family of growth factors are the most common and most well-studied drivers of EndMT, thus the induction of EndMT can be broadly split into TGF-β and non-TGF-β pathways (22, 23, 31–33). The TGF-β pathway of EndMT itself can be subdivided into canonical and non-canonical pathways. The canonical pathway is mediated by Smad2/3, while the non-canonical pathway is Smad2/3-independent and can act through a variety of other signal transducers such as mitogen-activated protein kinase (MAPK), phosphatidylinositol 3-kinase (PI3K), and PKC-δ (22, 23, 31–35). Non-TGF-β pathways of EndMT include Notch, Wnt, endothelin-1 (ET-1), and inflammatory signaling (22, 23, 31–33). Though the exact mechanisms of these pathways are varied and have not been fully elucidated, most pathways ultimately converge through the regulation of transcription factors such as SNAI1 and TWIST to suppress the expression of EC markers and promote the expression of mesenchymal proteins (22, 23, 31–39) (**Figure 1**). All of these pathways have been implicated in diabetes-induced EndMT in one or more diabetic complication, though there has not been any studies simultaneously examining specific pathways across different diabetic complications (12, 24, 40–46). Once an EC has undergone EndMT, the EC-derived mesenchymal cell remains committed to the mesenchymal phenotype, even if the driving stimulus is no longer present.

### 1.3 Epigenetic regulation and EndMT

The persistence of the mesenchymal phenotype in EC-derived mesenchymal cells is dependent on changes in epigenetic regulation. Epigenetic regulation describes heritable phenotypical changes absent changes in the genomic sequence. Epigenetic regulation encompasses several processes, including DNA methylation, histone modification, and non-coding RNA (ncRNA)-associated gene regulation (47–49). DNA methylation involves the covalent attachment of methyl groups to nucleotides, most commonly to the 5^th^ carbon of the cytosine residue in a CpG pair, and generally results in transcriptional inhibition (47–50). Histone modifications involve covalent changes to the histone proteins that bind the DNA, leading to either activation or repression of the associated DNA regions (47–49, 51). Common histone modifications include methylation, acetylation, phosphorylation, SUMOylation, and citrullination; the specific effects of histone modifications typically depend on the type of modification, the amino acid residue that is modified, and the location of the modification (47–49, 51). ncRNA-associated gene regulation involves RNA molecules that are transcribed from DNA but not translated into proteins; ncRNAs can potentiate or disrupt gene expression both at and beyond the transcriptional level (47–49, 52). Long ncRNAs (lncRNAs, greater than 200 nucleotides in length) can form complex tertiary structures and interact with RNA-binding proteins, serving as scaffolds, guides, or decoys for protein complexes, and leading to activation or repression of target loci (53–55). microRNAs (miR, short ncRNAs roughly 22 nucleotides in length) regulate translational silencing of specific mRNAs through complementarity (56–58). Circular RNAs (circRNAs) are ncRNAs derived from mRNA transcripts but circularized *via* back-splicing. circRNAs can modulate the expression of their parental genes by a decoy for translational machinery or inhibitory miRNAs, they can also interact with other proteins in order to produce specific outcomes (59–63).

**Figure 2:**
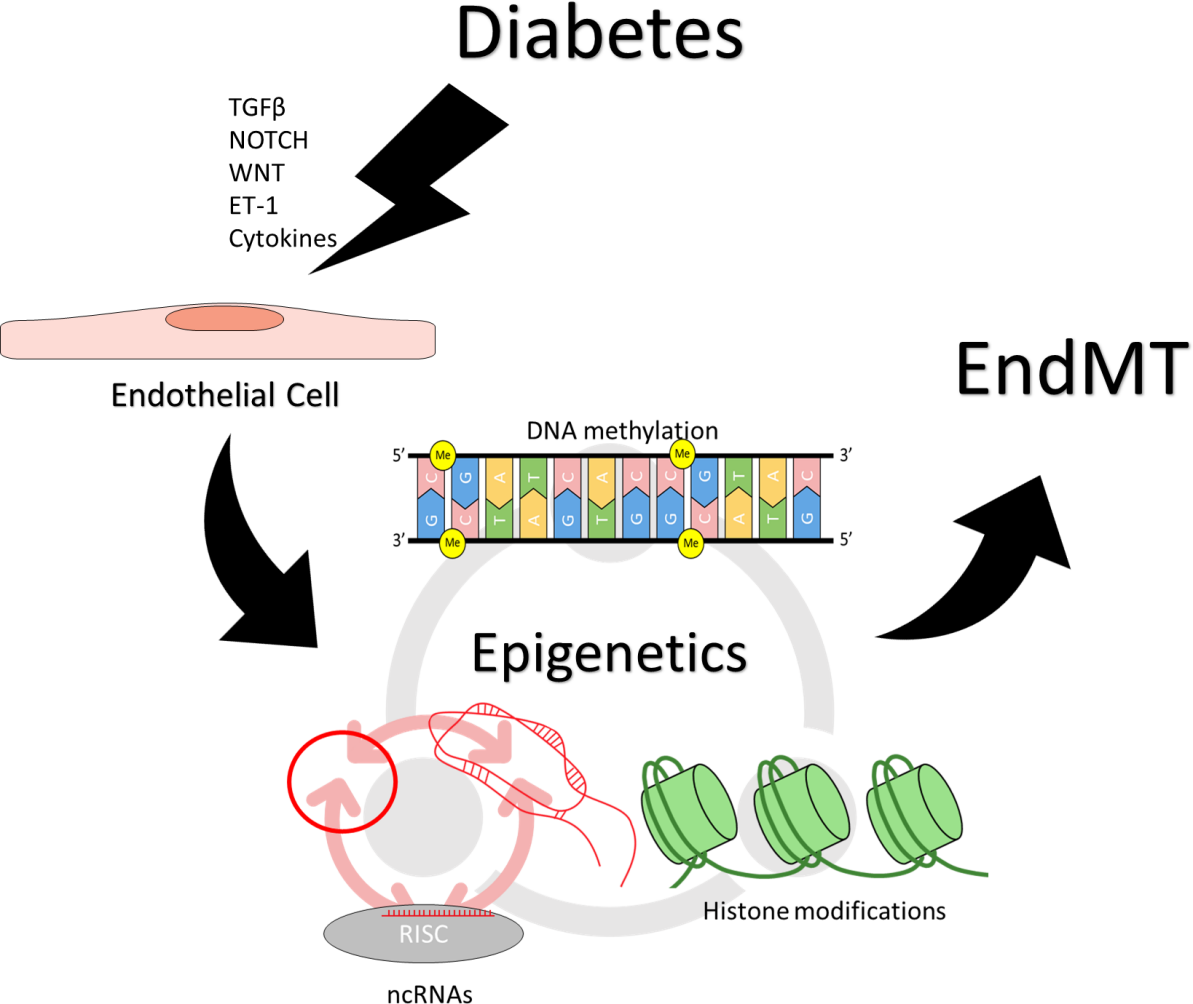
Epigenetic regulation of endothelial to mesenchymal transition. Induction and maintenance of EndMT is facilitated by changes in epigenetic regulation. Stimuli such as TGF-β, NOTCH, WNT, ET-1, inflammatory cytokines, which may result from hyperglycemia can effect changes in epigenetic regulation, resulting in persistently altered patterns of gene expression. Different epigenetic mechanisms also reciprocally regulate one another. DNA methylation, and histone modifications can influence the transcription of ncRNAs, while ncRNAs can regulate the proteins required for covalent modifications of DNA and histones. Different types of ncRNAs regulate also each other, with circRNAs and lncRNAs sponging miRNAs while miRNAs can target circRNAs and lncRNAs for degradation.

Epigenetic changes are involved at a variety of levels during EndMT, from modulating pro-EndMT signaling to enforcing long-term changes in endothelial and mesenchymal marker expression. Removal of DNA methylation has been shown to increase the expression of SNAI1 in epithelial cells (64). SNAI1 has been shown in a various cells to recruit histone deacetylases (HDACs) to target genes to suppress their expression (65, 66). HDAC9 has been found to mediate changes in endothelial and mesenchymal markers in EndMT, and targeted suppression of HDAC9 prevented EndMT in human coronary artery ECs (67). HDAC3α has been shown to promote TGF-β2 secretion, which acts as an autocrine inducer of EndMT (68). ncRNAs H19 and miR-200b have been found to modulate TGF-β signaling in the induction of EndMT (40–42). Beyond individual effects of each of the epigenetic mechanisms, epigenetic regulators also have reciprocal regulatory effects on one another. Covalent modifications to the DNA or the histones can influence the transcription of ncRNAs (56, 69–73). lncRNAs can inhibit miRNA activity by acting as a molecular sponge (53–55), or influence DNA and histone modification by guiding or obstructing the protein complexes (53–55, 74). miRNAs can inhibit the translation of proteins involved in DNA and histone modifications (75–77); they can also inhibit lncRNAs by inducing degradation (78–80). circRNAs can regulate DNA/histone modifying machinery and sponge miRNAs (59–62, 81). This creates an intricate network of regulation that underlies the process of EndMT, the precise balance of which determines the endothelial or mesenchymal nature of the affected cells (**Figure 2**).

## 2 EndMT in diabetic complications

Hyperglycemia is a known driver of EndMT in diabetes. High glucose-mediated endothelial damage activates a variety of EndMT-inducing pathways. High glucose promotes TGF-β signaling in ECs through the activation of the PKC pathway, which can trigger EndMT through both Smad-dependent and -independent pathways (12, 24, 40–42). The PKC pathway also triggers ET-1 production, which promotes EndMT independently from TGF-β (12, 43, 82). Furthermore, inflammatory responses triggered by ROS, AGE-RAGE and other hyperglycemia-induced pathways also contribute to EndMT in diabetes (12, 22, 39). Additionally, hyperglycemia effects changes in epigenetic regulation, and can increase the susceptibility of ECs to EndMT. Specific mechanistic links between hyperglycemia and EndMT have been reported in different diabetic complications organs (44, 83–87). Hyperglycemia and EndMT are closely intertwined in the early pathogenesis diabetic vascular complications, thus highlighting the importance of EndMT may provide new perspective and insight into the understanding of these complications.

### 2.1 Diabetic retinopathy and endothelial-tomesenchymal transition

Diabetic retinopathy is the leading cause of blindness in working-aged adults. Diabetic retinopathy is broadly divided into two stages, non-proliferative (NPDR) and proliferative (PDR) (9, 88–90). NPDR begins with asymptomatic glucose-induced changes in the retinal vasculature which compound and worsen, leading to increased vascular permeability, capillary non-perfusion, microaneurysms, microhemorrhages, neuronal damage, and intraretinal microvascular abnormalities (9, 88–90). The non-proliferative changes disrupt the perfusion of the retina, causing overexpression of angiogenic factors, leading to the characteristic growth of abnormal vessels, i.e., neovascularization in PDR (9, 88–90). Vision loss in diabetic retinopathy typically involves one of the following mechanisms: diabetic macular edema (DME), or hemorrhage and tractional retinal detachment (TRD) (9, 88–90). DME can occur during NPDR or PDR and is caused by fluid buildup as a result of leaky blood vessels (9, 88–90). TRD more limited to PDR and is caused by fibrovascular scarring of the abnormal vessels generated during neovascularization (9, 88–90). EndMT occurs throughout NPDR and PDR, and may contribute to either mechanism of vision loss in diabetic retinopathy.

ECs in the retina play an important role in maintaining the blood-retinal barrier (BRB), a highly selective barrier that keeps the retinal environment separate from general circulation (91, 92). Losing endothelial characteristics through EndMT disrupts the barrier function of these retinal ECs and results in increased permeability. Furthermore, increased deposition of matrix proteins such as type I collagen, type IV collagen and fibronectin due to the gain of mesenchymal traits may lead to thickening of the basement membrane, which also contributes to increased vascular permeability in diabetic retinopathy (40, 93). Increased vascular permeability not only contributes to risk of DME, but also allows circulating factors such as inflammatory mediators to enter the retina, leading to inflammation and further damage (91, 92). Breakdown of the BRB is a well-documented part of diabetic retinopathy and has many contributors, but EndMT does appear to play a substantial role. Inducing EndMT in non-diabetic mice has been shown to result in similar retinal vascular leakage levels to diabetic mice (42). Apart from disrupting the barrier function of the BRB, EndMT also contributes to other harmful pathways within diabetic retinopathy. EC-derived mesenchymal-like cells contribute to the population of myofibroblasts that participate in fibrosis and mediate TRD during PDR (94).

EndMT in diabetic retinopathy has been verified to be mediated by a variety of pathways. High glucose induced the suppression of two lncRNAs, MEG3 and H19, which reportedly mediate EndMT in the retina through Smad-independent TGF-β signaling (42, 83). MEG3 inhibition was mediated by glucose-induced promoter hypermethylation, while the mechanism of H19 has not been reported (42, 83). High glucose-induced suppression of miR-200b, facilitated EndMT in diabetic retinopathy through canonical TGF-β signaling (40). Experimentally induced overexpression of H19, MEG3 or miR-200b prevented EndMT by inhibiting MAPK, PI3K/AKT/mTOR, and Smad2 respectively, effectively cutting off TGF-β signal transduction (40, 42, 83). Other EndMT-inducing pathways are also at play in the diabetic retina. Notch2 is significantly upregulated in retinal ECs under high glucose conditions, and drove EndMT independently of TGF-β (44). Overexpression of miR-29a/b was able to suppress Notch2 and prevent EndMT (44). Differential expression of a variety of circRNAs has been reported in diabetic retinopathy and non-diabetic retinopathy animals, and circRNAs associated with EndMT-related processes were uniquely increased in animals with diabetic retinopathy (95).

### 2.2 Diabetic nephropathy and endothelial-to-mesenchymal transition

Diabetic neuropathy is the leading cause of end-stage kidney disease and kidney failure and has a strong association with cardiovascular morbidity and mortality (10, 96). Diabetic neuropathy is characterized by failure of the renal filtration system and progressive proteinuria (10, 96). The severity of diabetic neuropathy is correlated with the concentration of albumin in the urine (10, 96). Glomerular basement membrane (GBM) thickening is one of the earliest signs of diabetic neuropathy; despite becoming thicker, the GBM becomes disorganized and non-uniform, leading to passage of proteins from circulation into the filtrate (97). The passage of plasma proteins into the renal tubules, along with other high glucose-mediated events, such as mesangial expansion and generation of AGEs triggering inflammation and fibrotic changes within the glomeruli and renal tubules, resulting in glomerulosclerosis and tubulointerstitial fibrosis respectively (98–102). Inflammatory and fibrotic changes within the kidney impede kidney function, characterized by reduction of the glomerular filtration rate (GFR) (10, 97). The progressive decline in kidney function and GFR ultimately results in renal failure (10, 96, 97).

ECs of the kidney, somewhat similarly to those of the retina, have a role of maintaining a selective barrier—the glomerular filtration barrier (GFB) (103). EndMT negatively impacts the functional capabilities of the GFB by reducing endothelial junctional protein expression and increasing ECM protein deposition (85, 103, 104). Furthermore, EndMT in the glomerular ECs influences epithelial-to-mesenchymal transition (EMT) in adjacent epithelial cells, otherwise known as podocytes, which are also important supporters of the GFB (105). Thus, glomerular EndMT is closely related with podocyte EMT, which contributes to podocyte loss and further breakdown of the GFB (106). Inhibition of EndMT in glomerular ECs has been shown to reduce hyperpermeability, and inhibition of EndMT in diabetic mice has been shown to reduce albuminuria (85, 104). Additionally, EndMT in the kidney, as is the case in other organs, is a significant source of myofibroblasts and a contributor to sclerosis and fibrosis (28, 107, 108). One study has found that up to 30% of myofibroblasts found in the renal interstitium were of endothelial origin (8).

EndMT in diabetic neuropathy, as it is in most cases, has been shown to involve TGF-β-mediated responses. Rho-associated kinase (ROCK) is an effector of TGF-β that is upregulated under hyperglycemic conditions and promotes EndMT in diabetic neuropathy (85, 104). Suppression of ROCK *via* upregulation of miR-497 was able to attenuate EndMT in glomerular ECs (104). Canonical TGF-β signaling is also involved; different researchers have found that inhibition of Smad3 prevented EndMT in diabetic neuropathy (109, 110). One group showed direct blockade of Smad3 using a Smad3 inhibitor prevented AGE-induced EndMT, while another group showed that induction of miR-29 using the drug linagliptin prevented EndMT by inhibiting Smad3 phosphorylation. Non-TGF-β pathways have also been reported to occur in diabetic neuropathy. The pattern recognition receptor NOD2 has been shown to promote EndMT in response to high glucose by bypassing TGF-β receptors and directly activating MAPK (111). The serine protease inhibitor α2-antiplasmin similarly bypasses TGF-β and induces Smad2/3 activation *via* AGE-induced responses (112).The SET domain-containing protein 8 (SETD8) regulates EndMT in diabetic neuropathy by directly regulating SNAI1; upregulation of SETD8 suppressed SNAI1, and prevented EndMT (86). And signal transducer and activator of transcription 5A (STAT5A) has been reported to modulate latrophilin and seven transmembrane domain containing 1 (ELTD1) in order to regulate EndMT *via* a not well-characterized mechanism (113, 114). Lastly, on the chromatin level, methylation of histone 4 lysine 20 (H4K20me1) by lysine methyltransferase 5A has been found to inhibit EndMT, while trimethylation of histone 3 lysine 4 (H3K4me3) by Set1 has been found to induce EndMT (115–117).

### 2.3 Diabetic neuropathy and endothelial-to-mesenchymal transition

Patients with diabetes are 15-30 times more likely to require a lower limb amputation than non-diabetics (118, 119). The associated loss of sensation in diabetic distal somatosensory neuropathy renders patients especially susceptible to foot ulcers and infected wounds which may result in amputations (120, 121). Approximately one half of people with diabetes will develop neuropathy throughout their life (122). Diabetic neuropathy manifests as both numbness and pain, first beginning in the distal extremities. One common occurrence is paradoxical numbness and elevated pain sensitivity (123). This numbness and pain lead to reduced mobility and results in patients being more susceptible to falls, further exacerbating the risks of disabling injury (124). Currently, diabetic neuropathy is only treated through glucose control and pain management, as there is no effective cure for most diabetic complications.

The pathogenesis of diabetic neuropathy was initially understood as having a neurological basis, however, recent understandings of diabetic neuropathy now better characterize its pathogenesis as being primarily vascular in nature (125–128). Vascular dysfunction is the initial and underlying cause of all diabetic complications, and this remains consistent in diabetic neuropathy. The vascular supply to peripheral nerves is limited and blood flow can be easily compromised, any damage to the vasculature surrounding peripheral nerves rendering peripheral nerves vulnerable to ischemia (129). While no research has yet been done on the prevalence of EndMT in diabetic neuropathy, microvessels of the neural vasculature show both thickened basement membranes and disrupted ECs, indicating the possibility for EndMT to have occurred (130). There is still much to be discovered in diabetic neuropathy and elucidating the potential role of EndMT in the peripheral neuronal vasculature are needed to fill the gaps in understanding.

### 2.4 Diabetic cardiomyopathy and endothelial-to-mesenchymal transition

Cardiovascular disease is the leading cause of death among people with diabetes as diabetes is commonly comorbid with various other cardiovascular diseases such as atherosclerosis, coronary artery disease and hypertension. However, independent of these comorbid risk factors, diabetic cardiomyopathy is defined as abnormal form and function of the heart driven solely by diabetes and high blood glucose, which exists independently of other cardiac risk factors (131–133). Although other mechanistic changes may also play specific roles, microvascular pathology remains the major contributor (131, 134, 135). Patients with type 1 diabetes who do not have hypertension or coronary artery disease suffer higher rates of cardiac dysfunction than non-diabetic cohorts (136). Studies have found that left ventricular dysfunction is associated with diabetes when controlled for coronary artery disease and other heart diseases (137, 138). Additionally, the development of and risk for heart failure is directly correlated with blood glucose levels, with each 1% increase in glycated hemoglobin being linked to increased risk of heart failure in T1DM and T2DM patients (139). The pathogenesis of diabetic cardiomyopathy is not clearly defined as the heart is affected in numerous ways in diabetes, however diabetic cardiomyopathy is generally characterized by left ventricular hypertrophy and a reduction in cardiac contractility and function.

Fibrosis is one characteristic of diabetic cardiomyopathy, it results in imbalanced extracellular matrix protein production, leading to cardiac remodeling and impaired cardiac function, as the adult heart lacks regenerative abilities, cardiac fibrosis is difficult to reverse (140). In cardiac fibrosis as with other fibrotic diseases, abhorrent stimulation of fibroblasts to activate produces an overabundance of extracellular matrix proteins, leading to interstitial fibrosis and a thickened basement membrane. EndMT contributes to the pool of activated cardiac myofibroblasts, lineage tracing has found that 20-35% of cardiac fibroblasts had an endothelial origin (141, 142). Hyperglycemia is a potent cause of EndMT as high levels of glucose damage ECs and result in signaling derangement, resulting in ECs differentiating into a mesenchymal phenotype (143). Oxidative stress triggered by hyperglycemia drives the differentiation of ECs through the TGF-β1 and TGF-β2 pathways (144). Oxidative stress induces TGF-β1 and TGF-β2 signaling, which results in a reduction of endothelial markers and an increase in fibrotic markers and ECM proteins (145, 146). In addition, TGF-β further contributes to the development of fibrosis through the promotion of if ALK5/Smad3/NF-κB pathway as well as through aberrant activation of the Ras-GTPase pathway (141, 145–147). Receptor for advanced glycation end products (RAGE) is the receptor of advanced glycation end products (AGEs), both of which are increased in diabetes (148, 149). Knocking out RAGE is able to reduce the degree of EndMT and alleviated cardiac fibrosis in mice (149). As the transcriptional and post-transcriptional level, epigenetic alterations may play a major role in the mediation of EndMT.

EndMT in the heart was first discovered to be involved in the development of heart valves at an embryonic stage (150). Control of EndMT in the heart is epigenetically regulated and the persistent and heritable nature of epigenetic changes contributes to diabetic metabolic memory. Epigenetic modifications take the form of histone modifications, DNA methylation and through ncRNA mediation. During development, EndMT is terminated through the actions of HDAC3 (histone deacetylase 3), which results in the recruitment of EZH2 (enhancer of zeste homolog 2) to silence TGF-β1 (151). Though no mechanistic link has been made between histone modifications and EndMT in the context of diabetes, HDAC3 has been found to be significantly increased in T2DM patients and HDAC3 mRNA levels were positively correlated to poor glycemic control (152). HDAC3 being both associated with the termination of EndMT while being upregulated in diabetes is an interesting avenue of future studies. DNA methylation is the presence of methyl groups on cytosine bases in CpG islands in DNA (50). Addition of methyl groups *via* DNA methyltransferases results in stable gene silencing (50). In various models of cardiac fibrosis, the gene RASAL1 (Ras protein activator like 1, a Ras-signaling inhibitor) has been shown to be hypermethylated (147, 153). Methylation of RASAL1 promoter results in increased Ras-GTP activity, resulting in EndMT (153). Noncoding RNA are a class of epigenetic molecules with novel importance. So far, various microRNA have been discovered to be important in the context of cardiac fibrosis. miR-126-3p was found to be downregulated in HUVECs undergoing EndMT and over-expression of miR-126-3p was about to maintain ECs in an endothelial phenotype (142). Through regulation of the Wnt/β-catenin pathway, miR-222 is able to inhibit EndMT in mouse cardiac endothelial cells, and overexpression of miR-222 in diabetic mice reduces cardiac fibrosis (45). Transgenic mice that overexpressed miR-200b were found to have better cardiac function and reduced EndMT in cardiac tissue (41). miR-21, in contrast, was found to be upregulated in relation to EndMT, and inhibition of miR-21 improved cardiac function (46). Long noncoding RNA (lncRNA) have also been investigated as epigenetic regulators of EndMT. ANRIL regulates diabetic cardiomyopathy in concert with p300 and EZH2 of the PRC2 (polycomb repressive complex 2) complex and the hearts of diabetic ANRIL-knockout mice had reduced levels of ECM (154). LncRNA also interact with microRNA, for example, miR-9-5p interacts with the lncRNA ZFAS1 to mediate cardiac fibrosis in diabetic cardiomyopathy (155). Various other epigenetic modifications, including up- and down-regulated circRNAs have been observed in the diabetic heart which result in increased fibrosis, cardiac remodeling, and heart failure (95). Further investigations into the extent to which epigenetic modifications occur in EndMT in diabetic cardiomyopathy could lead to better and longer-lasting therapies for diabetic complications.

### 2.5 Endothelial-to-mesenchymal transition and macrovascular complications of diabetes

Atherosclerosis is the premier macrovascular complication of diabetes. It is thought to arise from chronic inflammation and injury to arterial walls, leading to accumulation of plaque which causes arteries to narrow and restricting blood flow (156). It is the cause of coronary artery disease, the most common heart disease in the US and Canada (157). Endothelial dysfunction lies at the heart of atherosclerosis. Hyperglycemic damage to the macrovasculature, as in the microvasculature, results in signaling derangement that leads to EndMT through the generation of reactive oxygen species and inflammatory cytokines (156, 158). Reactive oxygen species induce NF-κB signaling, triggering inflammation, promoting the accumulation of lipids and the formation of a fatty streak—the genesis of plaque (159). The mesenchymal cells derived from EndMT are critical in the progression of atherosclerosis. They secrete proinflammatory signaling molecules and produce and deposit ECM proteins that serve as scaffolding for the forming plaque (160). Recent search has delineated a substantial endothelial origin for mesenchymal cells of the arterial intima, with up to 30% in mice (156). Several pathways activated as a result of hyperglycemia all enhance TGF-β signaling, which directly induces the progression of EndMT (161). Signaling of TGF-β through ALK5 causes the activation of SMAD2/3 which results in the transcription of Snail, Slug and Twist, which contribute to the induction of EndMT and atherosclerosis development (87, 162–166). Various macrovascular complications are comorbid with diabetes. Individuals with T2DM are often also obese and suffer from hypertension. Hypertension, a common comorbidity of T2DM, further activates TGF-β signaling through Smad, exacerbating atherosclerosis.

Epigenetic modifications are also associated with atherosclerosis. PRC2 which is responsible for the repressive H3K27me3 histone modification has been found to be upregulated in the endothelium of blood vessels which are susceptible to atherosclerosis (167, 168). ECs isolated from human plaque show upregulated H3k27me3 when compared to ECs in regions without plaque (169). High levels of the histone modification H2K4me3 (histone 3 lysine 4 tri-methylation) has been detected in the ECs of rat aortas exposed to hyperglycemia, which resulted in enriched expression of Notch and development of a mesenchymal-like phenotype (117). EZH2 has been found to be upregulated in atherosclerosis as well as in ECs treated with high glucose (168–170). miR-10a is regulated by NF-κB signaling pathway and has been found to be downregulated in ECs in athero-susceptible regions compared to elsewhere, and low levels of miR-10a in the serum is associated with human atherosclerosis (171).

Although current studies have yet to concretely establish an epigenetic basis in EndMT in atherosclerosis, various epigenetic modifications that have been found in atherosclerosis are suggestive of EndMT pathways. For example, the previously mentioned miR-126 which is has been previously mentioned to be regulated in EndMT in cardiac fibrosis has also been found to be a regulator of the development of atherosclerosis in the coronary and aortic endothelium. MiR-126 inhibits VCAM-1 (vascular cell adhesion molecule 1), which in atherosclerosis interacts with inflammatory molecules to drive the formation of lesions (172). Atherosclerosis is a disease that exists outside of diabetes, and further research done on the potential role of EndMT may provide novel avenues for treatment of an incredibly prevalent disorder.

## 3 Molecules targeting EndMT in diabetic complications

A wide variety of pathways have been implicated in the pathogenesis of hyperglycemia-induced EndMT, but contributions of each pathway and the interplay between pathways in the regulation of EndMT is not fully understood. For example, inhibition of one of Notch, canonical TGF-β, or non-canonical TGF-β signaling alone can prevent EndMT in diabetic retinopathy, raising the question of if and how these pathways overlap and are co-regulated. Despite these gaps in understanding, there has been a wealth of research into the influences of various molecules on diabetes-induced EndMT, ranging from pharmaceutical agents which directly target EndMT-inducing pathways to miRNAs which act in an epigenetic manner. Most of these molecules have been identified in organ-specific research, however, as the molecules target common pathways, the findings may be applicable to diabetic complications in other organs as well.

In a case of having the cart before the horse, anti-diabetic drugs dapagliflozin, liraglutide, and linagliptin, which have long been used to aid in the regulation of blood glucose levels, have been shown to inhibit EndMT in of diabetic animals (110, 173, 174). The anti-EndMT effects of these drugs do not appear to be due to direct reduction of serum glucose levels, rather, dapagliflozin and liraglutide act by activating the AMP-activated protein kinase (AMPK), which attenuates intracellular TGF-β signaling (173–175), and linagliptin induces miR-29 to suppress Smad3 phosphorylation in TGF-β signaling. Further up the regulatory chain of hyperglycemia-induced EndMT, dietary supplements resveratrol and eicosapentaenoic acid have been shown to prevent EndMT in retinal and glomerular ECs respectively, by inhibiting PKC, thereby preventing the induction TGF-β and ET-1 (12, 24, 82, 176, 177). Short interfering RNAs (siRNAs) targeting pro-EndMT genes and lncRNAs, or those that mimic anti-EndMT miRNAs highlighted throughout the previous sections may also be viable approaches to preventing EndMT (178). Experimental silencing of lncRNAs ZFAS1 and MALAT1, and experimental induction of miRNAs 9, 29, 126, 145, 200b, 222, and 497 have proven to be potent suppressors of hyperglycemia-induced EndMT through various pathways (41, 44, 45, 104, 142, 155, 179, 180). Alternatively, synthetic lncRNAs might also be an option to suppress glucose-induced EndMT (181). Experimental upregulation of lncRNAs inhibited by glucose, H19 and MEG3, have been shown to prevent EndMT in diabetic retinopathy (42, 83).

## 4 Conclusion

For the millions of people living with diabetes, diabetic complications are almost an inevitable cause of morbidity and mortality. Vascular dysfunction lies at the nexus of diabetic complications. The manifestations of diabetic complications vary throughout the body and their regulatory components are different as well. In this review, we have summarized many of the myriad pathways which converge to mediate endothelial-to-mesenchymal transition as well as the epigenetic regulations which maintain EndMT. Though the connection between diabetes and EndMT has been established in diabetic nephropathy, cardiomyopathy and retinopathy, the causational relationship has yet to be experimentally observed in peripheral neuropathy. However, much has yet to be discovered and novel mechanisms have yet to be explored for their therapeutic potential. Considering that diabetic complications have no current treatment aside from symptom management and glycemic control, the discovery of therapies that may reverse or stop the progression of diabetic complications would make a huge impact on the lives of those suffering from the life-altering morbidities resulting from diabetic complications.

## Author contributions

EW and HW are equal contributors in writing the manuscript and the designing of figures. SC participated in topic design, manuscript editing and providing instructional support. All authors contributed to the article and approved the submitted version.
